# Application of OXITEST for Prediction of Shelf-Lives of Selected Cold-Pressed Oils

**DOI:** 10.3389/fnut.2021.763524

**Published:** 2021-10-21

**Authors:** Chieh-Hsi Tsao, Chih-Wei Chang, Yu-Chi Ho, Yung-Kun Chuang, Wei-Ju Lee

**Affiliations:** ^1^School of Food Safety, Taipei Medical University, Taipei, Taiwan; ^2^Crop Improvement Section, Taoyuan District Agricultural Research and Extension Station, Council of Agriculture, Executive Yuan, Taoyuan, Taiwan; ^3^Master Program in Food Safety, Taipei Medical University, Taipei, Taiwan; ^4^Research Center of Food Safety Inspection and Function Development, College of Nutrition, Taipei Medical University, Taipei, Taiwan

**Keywords:** OXITEST, oxidative stability, shelf-life, expeller-pressed oil, induction period, unsaturated fatty acid, multiple regression model

## Abstract

**Introduction:** Due to the enhanced awareness of consumers concerning healthy foods, homemade expeller-pressed oils have become popular worldwide. However, an extended storage period may lead to oxidization of the oil and exposure to hazardous byproducts by consumers.

**Methods:** In this study, 10 pressed oil samples prepared from common oilseeds using a small-scale expeller oil press were analyzed by OXITEST with a sample amount of 5 g of oil and an oxygen pressure of 800 kPa under accelerated conditions for shelf-life projections. The oil properties were investigated, including the recovery, smoke point, acid value, iodine value, “fatty acid composition, and contents of pigments and tocopherols”.

**Results:** The autoxidation reaction of various expeller-pressed oils under an accelerated testing system followed zero-order Arrhenius kinetics (*R*^2^ > 0.99). Shelf-lives of the pressed oils at 25°C were estimated by extrapolation to range 105~1,089 days. The obtained shelf-lives were significantly correlated with log induction period (IP) values (*r* > 0.81, *p* < 0.05) and unsaturated fatty acids (UFAs) (*r* < −0.69, *p* < 0.05), but not with the iodine value, acid value, or smoke point. Scatter diagrams between shelf-lives and UFAs suggested that these pressed oils could be grouped by two linear regression curves (*r* > 0.98, *p* < 0.05). The predictive equations using multiple linear regression are presented herein, with predictor variables of UFAs and an unspecified item involving potential influencing factors such as tocopherol contents (*r* > 0.88, *p* < 0.05).

**Conclusions:** Our findings first revealed that the UFA portion was partially correlated with the shelf-lives of selected expeller-pressed seed oils as estimated by the OXITEST. The derived equations can be applied for shelf-life predictions of expeller-pressed oils stored under dark ambient conditions based on the fatty acid profile.

## Introduction

Edible oils and fats are often acquired by mechanical pressing or solvent extraction followed by a refining process. Nowadays, the awakening of health consciousness in the general public has fostered the increasing popularity of mechanically pressed oils. Generally, pressed oils are processed without involving organic solvents and under a low temperature, possessing advantages of a low production of heat-induced chemical contaminants (1–3), preservation of more-beneficial minor components (4), and protection of unique flavors and odors for wide applications (5). Cold-pressed oils may be clarified by washing with water, settling, filtering, and centrifuging (6); however, they do not undergo a refining process of purification to eliminate minor compounds that are considered impurities and can potentially destabilize the oil (e.g., free fatty acids) or enhance oxidative stability (e.g., tocopherols) (7). Thus, safety concerns may arise from unintentionally consuming oxidized pressed oil, since it is not properly labeled and its shelf-life has not been evaluated. A suitable approach for estimating expeller-pressed oil shelf-lives is of importance to consumers to protect against ingesting oxidized oils and fats.

Unlike refined oils whose oxidative stability is mainly influenced by the fatty acid composition (8), the oxidative stability of pressed oils can be affected by several factors, including the fatty acid composition (9), natural antioxidants (10–12), prooxidants (13), and storage conditions (14). The existence of a correlation between tocopherols and fatty acids in vegetable oils has been discussed but still considered non-causal (15). Though the type of fatty acids is critically important for the oxidative stability of edible oils, substantial deviations were observed from the expectation that the higher the content of unsaturated fatty acids the lower the corresponding oxidative stability (6). Nevertheless, the negative trend of correlations between degree of unsaturation and oxidative stability was found to be absolute in previous studies (6, 15). Furthermore, the relative contribution of natural compounds to oxidative stability has proved elusive in edible oils and fats (6). In some cases, the seed-roasting process can significantly improve the oxidative stability of pressed oils by increasing antioxidant levels (11, 12), while extra virgin olive oil (EVOO) is rich in polyphenols, which are heat-sensitive natural antioxidants (10). Prooxidants such as chlorophylls can impair the stability of pressed oils. Pistachio oil, for example, has extremely low photo-oxidative stability due to the high contents of chlorophylls (13). Based on Taş and Gökmen's (16) literature review on total phenolic contents of various unroasted seeds including almond, peanut, pecan, pine nut, and walnut, similar low levels were found in those nuts without thermal treatment. Since expeller-pressed seed oils which are not subjected to a roasting process are regarded as cold-pressed oils, their oxidative stabilities are hypothesized to be mainly influenced by the fatty acid composition and endogenous antioxidants like tocopherols.

Oxidative stability is often expressed as an induction period (IP), which is the period of time before a dramatic increase in the rate of lipid oxidation begins (17). There are several methods for evaluating the oxidative stabilities of lipids. Generally, samples are subjected to accelerated and standardized oxidation tests, including but not limited to the Schaal oven test, oxygen bomb method, active oxygen method (AOM), oxidative stability index (OSI, also called the Rancimat method), and OXITEST, all of which apply high temperatures (18). Among them, the OXITEST method (AOCS Cd 12c-16) directly detects changes in oxygen pressure inside a chamber and automatically determines the IP which avoids possible operative errors (19). Moreover, since oil storage conditions (e.g., light-preventing packaging material and oxygen exposure) can significantly affect the shelf-life (14, 20, 21), the OXITEST system provides an ideal environment where light and oxygen exposures are well-controlled.

Currently there is no available standardized procedure for evaluating the shelf-life of pressed oils. Different shelf-life prediction models have been proposed to simulate EVOO degradation because of existing standards established by the Codex Alimentarius (22). For other pressed oils, the peroxide value (POV), p-anisidine value (p-AV), and sensory evaluation are occasionally used to monitor quality changes and determine IPs during accelerated storage tests (23, 24). The shelf-lives of edible oils used to be estimated by plotting the log IP vs. elevated temperatures with satisfactory *R*^2^ values of regression curves and extrapolation to ambient temperature to obtain the shelf-life (25–27). Previous studies attempted to anticipate the shelf-life based on the IPs of edible kernels obtained by OXITEST (28, 29). Further research is warranted to better characterize the OXITEST method in shelf-life estimation for a wide variety of oil types and its correlation with physicochemical properties.

This study aimed to explore the potential use of the OXITEST as a tool to predict the shelf-life of expeller-pressed oils. Commercially available oilseeds in Taiwan were extracted by a small-scale, home-use oil expeller to prepare pressed oil samples. The effects of the sample amount and oxygen pressure on OXITEST IPs and suitable temperature ranges were first evaluated. Then, the IPs of pressed oils were determined at 70, 90, and 100°C by the OXITEST method, and extrapolated to generate ambient shelf-lives. The shelf-life results were correlated with the log IP, smoke point, acid value, iodine value, and ratio of saturated fatty acids (SFAs), unsaturated fatty acids (UFAs), polyunsaturated fatty acids (PUFAs), and the SFAs/UFAs ratio to develop predictive models. These properties are commonly available on product labels of pressed oils.

## Methods

### Preparation of Oil Samples

All seeds and nuts including almonds, black sesame seeds, white sesame seeds, camellia seeds, golden linseed, peanuts, pecans, pine seeds, pumpkin seeds, sunflower seeds, and walnuts were purchased from local retailers (Taipei, Taiwan). Seed was homogenized using a Moulinette^®^ kitchen blender (Moulinex, France) for 4 min. Oil was extracted with an SX-TB02 screw oil press (Oiling Company, New Taipei City, Taiwan). Prior to pressing, the outlet nozzle was preheated to 70°C for 8 min. Pressed oils were prepared by an initial pressing of the seeds and then a re-pressing of the cake from the first press. The oils were collected in centrifuge tubes and centrifuged at 3,000 relative centrifugal force (RCF) for 10 min. The supernatant was decanted to another centrifuge tube, filled with nitrogen, and stored at −80°C in the dark until being used.

Solvent extraction was undertaken to evaluate the oil content of different seeds. Seeds were first ground into a powder and dried at 50°C for 12 h. Then, 10 g of dry powder was combined with 80 mL of n-hexane. The mixture was ultrasonicated for 30 min and then filtered through Whatman no. 1 filter paper with suction using a Buchner funnel. The filtrate was removed from the extract using a rotary evaporator at 40°C. The resulting oil was weighed, and the oil recovery was calculated.

### The OXITEST Method

The OXITEST apparatus (OXITEST, Velp^®^ Scientifica, Usmate, Italy) was implemented according to AOCS method no. Cd 12c-16 (30). An oil sample was loaded into a hermetically sealed titanium chamber with a thermostat. The OXITEST reactor subjected the oil samples to an accelerated oxidative process via heating at 50~110°C and exposure to an oxygen pressure of 600~800 kPa. The instrument was controlled by original software (OXISoftTM, Velp^®^) that monitored the oxygen pressure. At the end of the test, the program automatically calculated the IP from the resultant oxidation curves by the two-tangent method, i.e., the time required to reach the starting point of oxidation, corresponding to a sudden change in the rate of oxygen consumption (19).

#### Selection of Parameter Values

An optimization step was implemented to find suitable settings for the sample amount, oxygen pressure, and temperature range. Golden linseed oil was oxidized at 70°C, at oxygen pressures of 600 and 800 kPa, and sample quantities of 5, 7.5, and 10 g. Then, the IPs of golden linseed oil and walnut oil were analyzed at 50~110°C, with an oxygen pressure of 800 kPa and a sample amount of 5 g.

#### Sample Measurement

The IPs of different expeller-pressed seed oils were analyzed by the OXITEST method. Oil samples at 5 g were loaded in the chamber with an oxygen pressure of 800 kPa at 70, 90, and 100°C.

### Shelf-Life Estimation

Shelf-lives of expeller-pressed oils were estimated using the obtained IP values from the OXITEST method at 70, 90, and 100°C. The equations of zero oxidation kinetics were calculated using the following equation, Log IP = Log IP_0_-k_0_t for different temperature values, where Log IP_0_ represents the initial Log IP value, t represents the temperature, k_0_ is a constant.

### Oil Properties

The smoke point and acid value were respectively determined using AOCS official methods no. Cc 9a-48 and Cd 3d-63 (30). Carotenoids and chlorophylls were respectively determined at 470 and 670 nm by spectrophotometer (TC-1800 MK II, Tokyo, Japan) with 5% of oil in n-hexane (w/v) (31, 32). The pigment contents were calculated according to Boujemaa et al. (32).

The fatty acid profiles were determined based on modified AOCS official method no. Ce 2-66 to prepare the fatty acid methyl esters (FAMEs) (30). Analysis was performed by use of gas chromatography (Agilent 7890B, Agilent, CA, USA) equipped with DB-Heavy WAX column (30 m × 0.25 mm i.d., 0.25 μm film thickness, Agilent, CA, USA) and a flame ionization detector. The flow rates for hydrogen and air were 30 and 300 mL/min, respectively. Hydrogen was used as a carrier gas with a flow rate at 4 mL/min. Nitrogen was used as makeup gas at a flow rate of 30 mL/min. The oven temperature was kept at 50°C for 2 min, then increased to 170°C at a rate of 24°C/min, held for 5 min, increased to 210°C at a rate of 3°C/min, followed by 230°C at a rate of 10°C/min (held for 10 min). The injection volume was 0.1 μL and the mode was splitless direct injection. The detector temperature was 235°C. The iodine value was directly calculated from the fatty acid composition according to AOCS official method no. Cd 1c-85 (30).

Tocopherol contents were analyzed by high-performance liquid chromatography-ultraviolet (HPLC-UV) based on AOCS official method no. Ce 8-89 with some modifications (30). In brief, HPLC system involved a Thermo ConstraMetric 3200 pump equipped with a Thermo SpectroMonitor 3200 UV/vis detector. A reverse-phase analytical column YMC-Pack ODS-AM (250 × 4.6 mm, 5 μm) (YMC CO., LTD., Japan) was used for separation. The mobile phase was 2% (v/v) methanol in ultrapure water (Milli-Q, Millipore) at a flow rate of 1.5 mL/min. The injection volume was 20 μL and the detection wavelength was 292 nm. Quantification was performed using external standard curves of α- and γ-tocopherol standards.

### Data Analysis

Three biological replicates were performed for oil preparation. Then, all analyses were performed in triplicate (*n* = 3), except for fatty acid composition, tocopherol contents, pigments, and oxidative stability (*n* = 2). Data are presented as the mean (*n* = 2) and mean ± standard deviation (SD) (*n* = 3). Statistical analysis involved the use of Windows Excel 2019 (Microsoft, Redmond, WA, USA). Significant differences (*p* < 0.05) were determined by a two-tailed, paired Student's *t*-test. A simple linear regression analysis and multiple linear regression model were used to respectively determine the univariate and covariate associations. Pearson correlation coefficients were calculated, and the significance of associations was evaluated at the *p* < 0.05 level.

## Results

### Oil Yields of Expeller-Pressed Oils

In order to acquire freshly produced genuine pressed oils for an oxidative stability investigation, different oil seeds in Taiwan were collected and extracted in the laboratory using a commercial expeller oil press. As shown in Supplementary Table 1, the range of oil recoveries from different seeds prepared by the screw press was 22.8~62.5%. Recoveries of most expeller-pressed oils were similar to those obtained by solvent extraction, except for camellia, peanut, and pine nut oils, which had significantly lower recoveries (*p* < 0.05) (Supplementary Table 1). Yields of pressed oils were calculated to be 56.4~104.5%. In the present study, pressed oils were prepared by initial pressing of seeds and re-pressing of the cake of first press to achieve satisfactory recoveries. Nevertheless, pine nuts and peanuts were more ductile due to their high moisture contents (~10%), resulting in less efficiency for oil separation from the seed residue and oil loss through the operation of the expeller press rod (31). On the contrary, low oil recovery of camellia seeds was probably due to the hardness of the seed. Camellia seeds are usually roasted prior to pressing to enhance oil extraction. Since the seed roasting process was proven to be a factor influencing camellia oil oxidative stability (33), it was excluded from our study design. These 10 selected seeds presented acceptable oil recoveries (>20%) when pressed by the small-scale commercial screw oil press, indicating that all had potential to be used as raw materials for homemade expeller-pressed oils.

### Physicochemical Properties of Expeller-Pressed Oils

The appearances of the expeller-pressed oils are presented in Supplementary Figure 1. Most of the oils were yellow at different saturations except for pumpkin seed oil. As shown in Supplementary Table 2, the values of carotenoids ranged from 0.51 to 10.08 mg/kg, while the values of chlorophylls varied between 0.07 and 21.81 mg/kg. Pumpkin seed oil appeared green and dark brown owing to the rich contents of chlorophylls and carotenoids, in agreement with Boujemaa et al. (32).

The fatty acid compositions of expeller-pressed oils are shown in Table 1. The predominant fatty acid was found to be oleic acid in almond oil (63.3%), camellia oil (78.3%), peanut oil (79.1%), and pecan oil (58.0%). The major fatty acids were linoleic acid and oleic acid in black sesame oil (44.3 and 38.0%), pine nut oil (45.1 and 28.0%), pumpkin seed oil (47.3 and 32.0%), and sunflower seed oil (52.0 and 36.6%), while linoleic acid, linolenic acid, and oleic acid were predominant in walnut oil. Golden linseed oil was abundant in PUFAs, particularly linolenic acid (54.4%). The fatty acid compositions of expeller-pressed oils mentioned above paralleled previous studies (34–40). All of the theoretical iodine values of pressed oils were <160 g I_2_/100 g oil, except for that of linseed oil (Table 2). The iodine values of these pressed oils ranged from 76.6 g I_2_/100 g for peanut oil to 187.2 I_2_/100 g for linseed oil, reflecting a significant variation in the overall degree of saturation (Table 1).

**Table 1:** Fatty acid compositions of 10 expeller-pressed oils.

**Oil type**	**C14:0**	**C16:0**	**C16:1**	**C17:0**	**C18:0**	**C18:1**	**C18:2**	**C18:3**	**C20:0**	**C20:1**	**C22:0**	**SFA**	**UFA**	**PUFA**
Almond oil	0.04	8.08	0.49	0.04	1.35	63.26	26.55	0.09	0.04	0.05	ND	9.55	90.44	26.64
Black sesame oil	0.02	10.93	0.12	0.03	5.82	38.01	44.33	ND	0.55	0.12	0.08	17.43	82.58	44.33
Camellia oil	0.04	10.49	0.12	0.04	1.82	78.30	8.79	ND	ND	0.39	ND	12.39	87.60	8.79
Golden linseed oil	0.36	4.68	ND	ND	3.81	21.50	15.27	54.39	ND	ND	ND	8.85	91.16	69.66
Peanut oil	0.02	7.41	0.03	0.09	3.45	79.12	4.91	ND	1.07	ND	2.39	14.43	84.06	4.91
Pecan oil	0.06	8.15	0.07	0.05	2.37	57.95	31.11	ND	0.07	0.17	ND	10.70	89.30	31.11
Pine nut oil	ND	6.22	0.08	0.04	2.59	28.04	45.13	16.09	0.35	1.39	0.07	9.27	90.73	61.22
Pumpkin seed oil	0.11	13.63	0.11	0.09	6.07	31.98	47.31	0.27	0.36	ND	0.06	20.32	79.67	47.58
Sunflower seed oil	0.06	6.75	0.09	0.03	3.38	36.61	51.97	0.2	0.18	0.12	0.60	11.00	88.99	52.17
Walnut oil	0.35	6.04	ND	ND	2.52	14.76	60.00	16.34	ND	ND	ND	8.91	91.10	76.34

*Results are expressed as the mean (n = 2)*.

*ND, not detectable. SFAs, saturated fatty acids; UFAs, unsaturated fatty acids; PUFAs, polyunsaturated fatty acids*.

**Table 2:** Smoke points, acid values, and iodine values of 10 expeller-pressed oils.

**Oil type**	**Iodine value** **(g I_2_/100 g oil)^a^**	**Smoke point (^°^C)**	**Acid value** **(mg KOH/g oil)**
Almond oil	101.1	174.8 ± 3.5	0.53 ± 0.07
Black sesame oil	109.7	172.8 ± 4.5	3.89 ± 0.08
Camellia oil	83.0	176.5 ± 3.5	1.37 ± 0.06
Golden linseed oil	187.2	134.3 ± 1.5	1.70 ± 0.07
Peanut oil	76.6	141.8 ± 2.8	1.06 ± 0.05
Pecan oil	103.9	193.7 ± 5.5	0.23 ± 0.01
Pine nut oil	145.5	165.8 ± 1.8	1.51 ± 0.05
Pumpkin seed oil	110.3	184.3 ± 4.2	2.27 ± 0.12
Sunflower seed oil	122.2	109.0 ± 5.1	1.85 ± 0.12
Walnut oil	159.4	148.3 ± 4.2	2.26 ± 0.07

*Results of the smoke point and acid value are expressed as the mean ± standard deviation (n = 3), while iodine values are expressed as mean (n = 2)*.

a *Calculated from the fatty acid compositions according to the AOCS Cd 1-85 method*.

The smoke points and acid values of the 10 pressed oils are shown in Table 2. Smoke points of the expeller-pressed oils ranged from 109.0 (sunflower seed oil) to 193.7°C (pecan oil). Unrefined oils tend to contain free fatty acids and other impurities, resulting in higher acid values and lower smoke points compared to refined oils (41). Acid values of the 10 seed oils were in a range of 0.20~3.89 mg KOH/g oil, all within the specification of <4 mg KOH/g oil set by the Codex Alimentarius Commission (CAC) for cold-pressed and virgin oils (42).

### Optimization of the OXITEST Method

The IPs obtained by the OXITEST with different weights of golden linseed oil and initial oxygen pressure settings at 70°C are shown in Table 3. The IPs of different weights (5, 7.5, and 10 g) of golden linseed oil with 600 kPa of oxygen pressure were in a range of 36.2~36.4 h. The effect of the oil amount on the IP in the OXITEST system was considered negligible, consistent with results of Comandini et al. (43) in that there were no significant differences among IPs using 3, 5, 7, or 10 g of sunflower oil at 110°C. The sample amount was optimized to 5 g in order to minimize the amount of sample wasted while maintaining an even distribution of oil on the sample plate and regularizing the oxygen exposure. On the other hand, the obtained IPs with the oxygen pressure set to 800 kPa were significantly shorter than those at 600 kPa (*p* < 0.05) (Table 3), indicating that the rate of lipid oxidation may increase under higher oxygen pressures. To reduce the required experimental time, the oxygen pressure parameter was adjusted to the upper limit of 800 kPa in the following experiments.

**Table 3:** Induction periods (IPs) of 5, 7.5, and 10 g of golden linseed oil determined by the OXITEST with oxygen pressures set to 600 and 800 kPa.

**Oxygen pressure**	**Oil weight**		
	**10.0 g**	**7.5 g**	**5.0 g**
600 kPa	36.3 ± 0.4^a^	36.4 ± 0.2^a^	36.2 ± 0.2^a^
800 kPa	33.8 ± 0.1^b^	34.5 ± 0.0^b^	34.6 ± 0.1^b^

*Results are expressed as the mean ± standard deviation (n = 3)*.

*Different lowercase letters in the same column indicate a statistical difference (p < 0.05)*.

The accelerated oxidation test was conducted in a temperature range of 50~110°C by the OXITEST reactor to investigate the linearity between log IP and temperature. The two pressed oils with the highest iodine values were tested. As shown in Supplementary Figure 2, linear correlations between log IP and temperature were good for walnut oil (*R*^2^ = 0.9992) and golden linseed oil (*R*^2^ = 0.9986). The IP of walnut oil at 110°C was 0.65 h (data not shown), which deviated from the regression curve and was therefore excluded. Similarly, Zhang et al. (44) analyzed the IP of *Paeonia ludlowii* kernel oil by the OXITEST at 70~110°C to extrapolate the shelf-life at room temperature. In that study, good linearity was found over a wide temperature range of 50~100°C, suggesting that the OXITEST method depending on oxygen consumption could be time-saving for shelf-life estimations since the oxidation mechanism was not altered at high temperatures (80~100°C).

### Oxidative Stability and Estimated Shelf-Life of Expeller-Pressed Oils

IPs and estimated shelf-lives of 10 pressed oils assessed by the OXITEST method are given in Table 4. The IP decreased as the storage temperature increased, while the shelf-life was extended when the temperature decreased from 25 to 20°C. A kinetic study was performed using OXITEST data to generate oxidation decay curves. As shown in Table 4, zero-order equations modeled the oxidation kinetics of the pressed oil samples at 70~100°C (*R*^2^ > 0.99). Shelf-lives of the pressed oils could therefore be predicted by extrapolation of the calibration curves at 70, 90, and 100°C owing to good linearity. The obtained rate constants (k_0_ values) known as temperature acceleration factors were in the range of 0.033~0.043 (Table 4). Although a poor correlation was found between rate constants and shelf-lives, a moderate positive correlation was observed between rate constants and iodine values (*r* = 0.67), indicating that the oxidation rate increased as the unsaturation degree was higher (data not shown). Peanut oil possessed the longest shelf-life with the presence of the highest log IP_0_ compared to all other oils, followed by sesame oil, pecan oil, and pumpkin seed oil, which also showed higher log IP_0_ values. Unlike rate constants, the obtained log IP_0_ values were highly correlated with shelf-lives (*r* = 0.90).

**Table 4:** Induction periods (IPs), Arrhenius equations, and estimated shelf-lives of 10 expeller-pressed oils by the OXITEST method.

**Oil type**	**IP (h)**			**Arrhenius equation^a^**			**Estimated shelf-life (days)**	
	**70^**°**^C**	**90^**°**^C**	**100^**°**^C**	**Log IP_**0**_**	**k_**0**_**	**Coefficient of determination** **(*R*^**2**^)**	**20^**°**^C**	**25^**°**^C**
Almond oil	85.00	20.62	8.83	4.2126	0.0325	0.9979	152	105
Black sesame oil	144.00	21.92	8.78	4.9952	0.0405	1.0000	638	400
Camellia oil	85.22	14.20	6.63	4.5294	0.0372	0.9985	254	166
Golden linseed oil	34.48	4.60	1.78	4.5457	0.0430	0.9998	202	123
Peanut oil	ND	81.52	33.62	5.3799	0.0385	1.0000	1,697^b^	1,089^b^
Pecan oil	151.00	25.08	10.11	4.9179	0.0391	1.0000	570	363
Pine nut oil	74.00	10.88	4.32	4.7509	0.0412	0.9999	352	219
Pumpkin seed oil	145.00	23.05	9.55	4.9211	0.0395	0.9999	564	358
Sunflower seed oil	62.60	10.32	4.62	4.4458	0.0379	0.9992	203	131
Walnut oil	44.47	6.50	2.67	4.5043	0.0409	0.9996	202	126

*Results are expressed as the mean (n = 2)*.

a *Zero-order Arrhenius equations (Log IP = Log IP_0_-k_0_t) are presented*.

b *The induction period (IP) of peanut oil was longer than 240 h, the instrumental upper limit of the IP determination. The IPs of peanut oil determined at 80, 90, and 100°C were further extrapolated to acquire the shelf-lives*.

In Table 4, shelf-lives at 25°C of expeller-pressed oils varied in a very wide range from ~3 months for almond oil to ~3 years for peanut oil at 25°C. Also, differences in shelf-lives estimated at either 20 or 25°C are signified in Table 4 in consideration of seasonal or regional adjustment. The order of shelf-life was peanut oil > black sesame oil > pecan oil ≈ pumpkin seed oil > pine nut oil > camellia oil > sunflower seed oil ≈ walnut oil ≈ golden linseed oil > almond oil, in line with those of IP values at all temperatures. Since seed oils are rich in UFAs, greater oxidative stability was associated with a higher ratio of monounsaturated fatty acids (MUFAs) (45). Verardo et al. (45) investigated the oxidative stability of walnut samples, revealing an existing positive correlation between OXITEST results and the MUFA content (*R*^2^ = 0.8921, *p* < 0.001) and a negative correlation between OXITEST results and the PUFA content (*R*^2^ = −0.8865, *p* < 0.001). The predominant fatty acid was oleic acid in peanut oil (79.12%), pecan oil (57.95%), and camellia oil (78.3%), which exhibited relatively superior oxidative stabilities and longer shelf-lives (Table 4). Exceptionally, almond oil was the most susceptible to oxidation even though the fatty acid composition of was rich in oleic acid (63.26%), which may be attributed to the contents and composition of tocopherols.

Tocopherol contents of pressed oils are presented in Supplementary Table 3. Tocopherols are antioxidants widely found in vegetable oils which can inhibit oxidation and therefore prolong the IP of those oils (15, 46). Generally, pressed seed oils are rich in tocopherols in either the α, γ, or both forms, and the antioxidant efficacy of γ-tocopherol is known to be higher than that of α-tocopherol (15, 46). Pecan oil, peanut oil, and golden linseed oil contained γ-tocopherol in appropriate high concentrations (52.8~60.3 mg/100 g oil), whereas lower γ-tocopherol levels were found in camellia oil, almond oil, and sunflower seed oil (10.1~16.5 mg/100 g oil). As a result, peanut oil and pecan oil were more oxidatively stable than almond, sunflower seed, and camellia oils during storage. Moreover, almond oil and sunflower seed oil possessed α-tocopherol at high concentration (>30 mg/100 g oil) which may exhibit prooxidant effects (15, 46, 47). As for golden linseed oil, low oxidative stability was mainly attributed to a high ratio of PUFAs (linolenic acid > 50%) in the fatty acid composition. Similar to linseed oil, pine nut oil, walnut oil, and sunflower seed oil were primarily composed of PUFAs (>50%) and contained low to moderate levels of γ-tocopherol (16.5~36.3 mg/100 g oil), possessing relatively shorter induction periods. Sesame oil which was also rich in PUFAs and abundant in γ-tocopherol showed long shelf-lives, ranking second only to peanut oil.

### Correlations Between Oil Properties and Shelf-Life

Linear regression analyses were conducted between shelf-lives and log IPs, smoke points, acid values, and iodine values of expeller-pressed oils. Pumpkin seed oil was excluded because the endogenous chlorophyll could significantly influence the shelf-life and cause a deviation from the other seed oils (32). As shown in Table 5, significant correlations were found between log IP values determined at different temperatures and shelf-lives (*p* < 0.05). However, it should be noted that since log IP values obtained by the OXITEST method were not totally parallel to shelf-lives, attention should be paid when comparing oxidative stability based on a single IP at one specific temperature. No significance was observed in the correlation analysis of the shelf-life with the smoke point, acid value, or iodine value (Table 5). Although the initial acid value may considerably contribute to the oxidative stability at high temperature (25), our results demonstrated that the shelf-lives of pressed oils could not be predicted by either the smoke point, acid value, or iodine value. It is necessary to estimate the shelf-life based on a currently accepted qualified method.

**Table 5:** Correlations of log induction period (IP) value, smoke point, acid value, “iodine value, and fatty acid compositions” with the shelf-life of nine expeller-pressed oils.

	**Correlation coefficient (** ***r*** **)**	
	**Shelf-life at 20^**°**^C**	**Shelf-life at 25^**°**^C**
Log IP at 70°C	0.9094^*^	0.9124^*^
Log IP at 90°C	0.8394^*^	0.8451^*^
Log IP at 100°C	0.8177^*^	0.8242^*^
Smoke point	−0.0197	−0.0192
Acid value	−0.0890	−0.0995
Iodine value	−0.5222	−0.5320
SFAs	0.5910	0.5881
UFAs	−0.6972^*^	−0.6951^*^
PUFAs	−0.5549	−0.5654
SFAs/UFAs ratio	0.6006	0.5975

* *Correlation was significant at the 0.05 level*.

*SFAs, saturated fatty acids; UFAs, unsaturated fatty acids; PUFAs, polyunsaturated fatty acids*.

**Figure 1:**
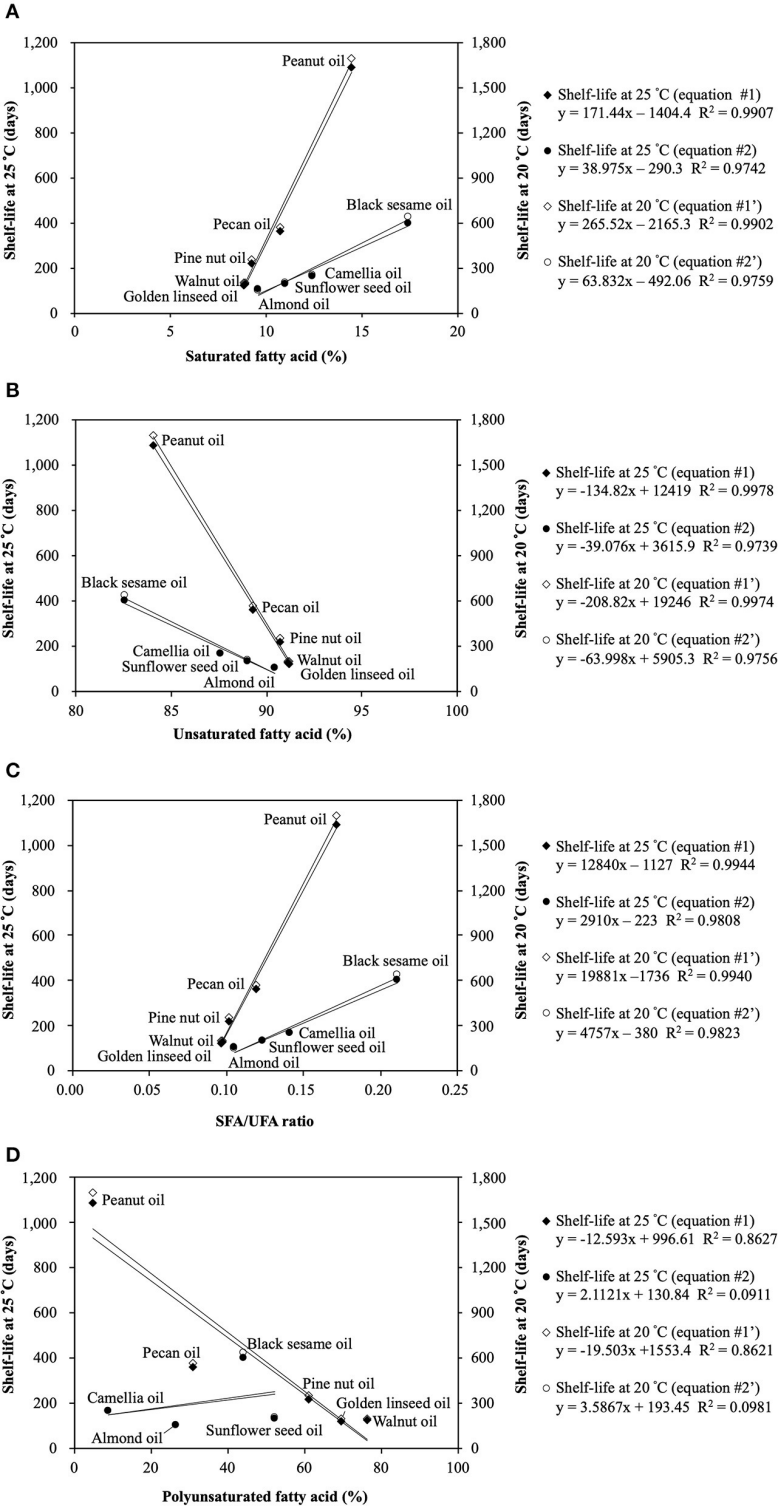
The two-curve models between fatty acid compositions and shelf-lives at 20 and 25°C: **(A)** Saturated fatty acid (SFA); **(B)** Unsaturated fatty acid (UFA); **(C)** SFA/UFA ratio; **(D)** Polyunsaturated fatty acid (PUFA).

As expected, the shelf-life was positively correlated with SFAs and the SFAs/UFAs ratio, while negatively correlated with UFAs and PUFAs (Table 5). A significant association was observed between UFAs and the shelf-life (*p* < 0.05), but not with SFAs, PUFAs, or the SFAs/UFAs ratio. Furthermore, these seed oils could be separated into two groups based on the linear regression results. Figure 1 presents the two-curve models, namely equations #1 and #2, between fatty acid compositions (SFAs, UFAs, SFAs/UFAs, and PUFAs) and shelf-lives at 20 and 25°C. SFAs, UFAs, and SFAs/UFAs showed higher coefficients of determination (*R*^2^) compared to PUFAs, and UFAs exhibited the highest degree of correlation (*R*^2^ > 0.98) with shelf-lives at 20 and 25°C. A multiple linear regression model approach was further adopted to simplify the equations, taking into account this new non-specific variable (X_2_) along with fatty acid compositional ratios (X_1_). As listed in Table 6, significant correlations were found for all regression curves between fatty acid ratios and shelf-lives at 20 and 25°C (*p* < 0.05), except for PUFAs. The shelf-life prediction model was established based on UFAs, because the correlation and significance were both stronger than for other variables (Figure 1). Moreover, the model was examined by testing the shelf-life of expeller-pressed white sesame seed oil. IPs of white sesame seed oil were determined to be 681 days at 25°C and 1,119 days at 20°C by the OXITEST method, which were similar to the predicted results of 692 days at 25°C and 1,084 days at 20°C based on UFAs, indicating that the prediction model was feasible and applicable. Expanding the applicability of this model to more types and species of oils requires further study in the future.

**Table 6:** Multiple regression analyses between the fatty acid composition and shelf-life of nine expeller-pressed oils.

**X_1_**	**Shelf-life at 20^°^C (Y)**			**Shelf-life at 25^°^>C (Y)**		
	**Equation^a^**	**Correlation coefficient (*r*)**	***p***	**Equation^a^**	**Correlation coefficient (*r*)**	***p***
SFAs	*y* = 142X_1_ + 600X_2_-1,474	0.8374	0.0266	*y* = 90X_1_ + 378X_2_-935	0.8291	0.0306
UFAs	*y* = −138X_1_ + 550X_2_ + 12,337	0.8953	0.0078	*y* = −88X_1_ + 347X_2_ + 7868	0.8885	0.0093
SFAs/UFAs	*y* = 10563X_1_ + 586X_2_-1,224	0.8376	0.0266	*y* = 6717X_1_ + 370X_2_-776	0.8292	0.0305
PUFAs	*y* = −14X_1_ + 512X_2_ + 773	0.7615	0.0741	*y* = −9X_1_ + 326X_2_ + 500	0.7666	0.0701

a *The equations where y is the shelf-life, X_1_ is parameters regarding fatty acid composition, and X_2_ is 0 for almond, camellia, sunflower, and sesame oils or 1 for peanut, pecan, pine nut, walnut, and golden linseed oils*.

*SFAs, saturated fatty acids; UFAs, unsaturated fatty acids; PUFAs, polyunsaturated fatty acids*.

The unspecified variable (X_2_) could be explained by the variation of tocopherol content and composition. Castelo-Branco et al. (48) proposed using the antioxidant capacity as a surrogate measure for the stability of vegetable oils. It was also reported that γ-tocopherol was the most important predictor in the models (48). Since tocopherols are efficient antioxidants at low concentrations but they gradually lose efficacy as their concentrations in the vegetable oils increase, defeating the purpose of correlating with shelf-life (31, 46, 47). In our study, the grouped pressed oils (i.e., linseed oil, walnut oil, peanut oil, and pecan oil fitted in equation #1) showing better resistance to oxidation with an increasing UFA ratio tended to contain higher levels of γ-tocopherol than the other group (i.e., almond oil, camellia oil, and sunflower seed oil fitted in equation #2) (Supplementary Table 3). Pine nut oil contained both γ-tocopherol and α-tocopherol at similar and appropriate levels which are supposed to elevate the antioxidant activity. An exception occurred in that sesame oil principally contained γ-tocopherol but was grouped in equation #2, which was likely due to the γ-tocopherol content being high enough (> 100 mg/100 g oil) to be prooxidant (15, 47). Since γ-tocopherol has a stronger antioxidant effect than α-tocopherol, the slope values of equation #1 appeared to be higher than those of equation #2.

Although correlations among tocopherols, polyphenols, and fatty acid compositions are still controversial (6, 15, 48), our findings demonstrated that the unsaturated degree was the most crucial factor for these selected homemade expeller-pressed seed oils. The forms and levels of tocopherols also play important roles in predicting the shelf-life of these seeds, consistent with our hypothesis that the major influencing factors for shelf-lives of expeller-pressed seed oils are the UFA ratio and antioxidants, mainly tocopherols.

## Discussion

Cold-pressed oils are frequently chosen by consumers due to their reputation as a good source of natural nutrients and ingredients from seeds. According to previous studies, the oxidative stability of pressed oils can be affected by multiple factors, such as the degree of saturation of fatty acids, minor compound constituents, the processing temperature, and storage conditions (8, 11, 14, 33, 49). In the present study, the oxidative stability of expeller-pressed oils was first studied using the OXITEST system to develop a prediction model in order to assess ambient storage shelf-lives. To achieve this aim, the oil type, processing conditions, and an oxidative stability evaluation method were restricted to decrease the impacts of confounding variables described below. First, the processes of drying and expeller-pressing for pressed oil preparation were consistent. Roasting effects were not addressed in this study, because thermal treatment may significantly enhance the oxidative stability of pressed oils (12, 33, 49). Second, the IPs measured by OXITEST were less likely to be affected by light, oxygen, and anthropic factors, since it automatically monitors changes in the oxygen pressure in a sealed chamber with software controlling the entire operation and calculating the IP. In addition, effects of the sample amount and operation temperature that ranged up to 100°C on the resultant shelf-life were insignificant based on our results, while the IPs became significantly shorter as the oxygen pressure increased from 600 to 800 kPa. Therefore, shelf-life prediction from the extrapolation of Arrhenius plots should be only applied to freshly prepared pressed oils stored in an opaque bottle with as low oxygen exposure as possible. Last, minor constituents like tocopherols and polyphenols in the pressed oils may increase the complexity of shelf-life predictions. Pumpkin seed oil was excluded as a notable exception from the prediction equation due to the occurrence of chlorophylls. For the other nine seeds, this study first revealed an unspecified variable (X_2_) parceled the latent impacts of other minor constituents mainly tocopherols in the final regression equation. Furthermore, the equations seemed to be applicable for predicting the shelf-life of different varieties of these seeds (i.e., black and white sesame seeds).

Herein, ambient storage shelf-lives of freshly prepared expeller-pressed oils were estimated by the OXITEST, and a prediction model was first established based on an association with the fatty acid composition. A significant correlation was found between the shelf-life and UFA ratio, suggesting that the fatty acid composition is one of the crucial factors in pressed oil stability. The scatter diagrams appeared to fit the multiple linear regression model with two predictor variables, where X_1_ was the UFA ratio and X_2_ was defined as 0 or 1 for different types of seeds (*R*^2^ > 0.98), which shed new light on prediction models of expeller-pressed oil shelf-life. Nonetheless, the current study is limited by the small sample size and restricted experimental conditions: roasting process, storage environment, and the compositional variances in oils such as polyphenol contents were not determined. Further research involving larger sample sizes should be done to elucidate other potential influencing factors.

## Data Availability Statement

The raw data supporting the conclusions of this article will be made available by the authors, without undue reservation.

## Author Contributions

C-HT: investigation and writing—original draft preparation. C-WC: investigation. Y-CH: resources. Y-KC: methodology. W-JL: conceptualization, funding acquisition, supervision, and writing—review and editing. All authors contributed to the article and approved the submitted version.

## Funding

This work was supported by Taipei Medical University (project no. TMU106-AE1-B39) and the Taiwanese Ministry of Science and Technology (project no. MOST108-2813-C-038-028-B).

## Conflict of Interest

The authors declare that the research was conducted in the absence of any commercial or financial relationships that could be construed as a potential conflict of interest.

## Publisher's Note

All claims expressed in this article are solely those of the authors and do not necessarily represent those of their affiliated organizations, or those of the publisher, the editors and the reviewers. Any product that may be evaluated in this article, or claim that may be made by its manufacturer, is not guaranteed or endorsed by the publisher.
