# Supplementary material for: Application of OXITEST for Prediction of Shelf-Lives of Selected Cold-Pressed Oils
Source: Front Nutr. 2021 Oct 21;8:763524. doi: 10.3389/fnut.2021.763524 (PMC8566681; doi:10.3389/fnut.2021.763524)
Supplement: Supplementary file 1 [file Data_Sheet_1.PDF]

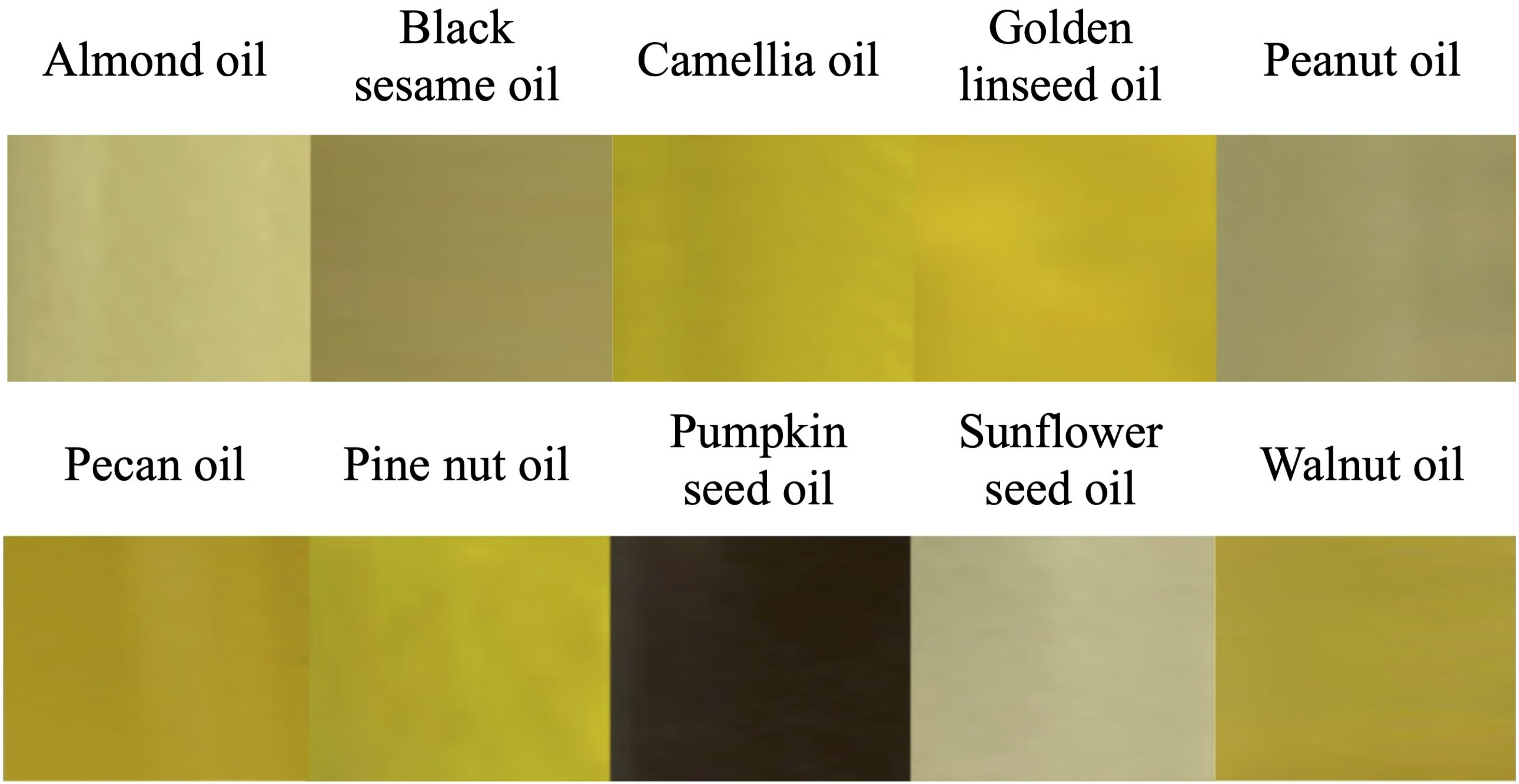

Supplementary Figure 1. Appearances of expeller-pressed oils

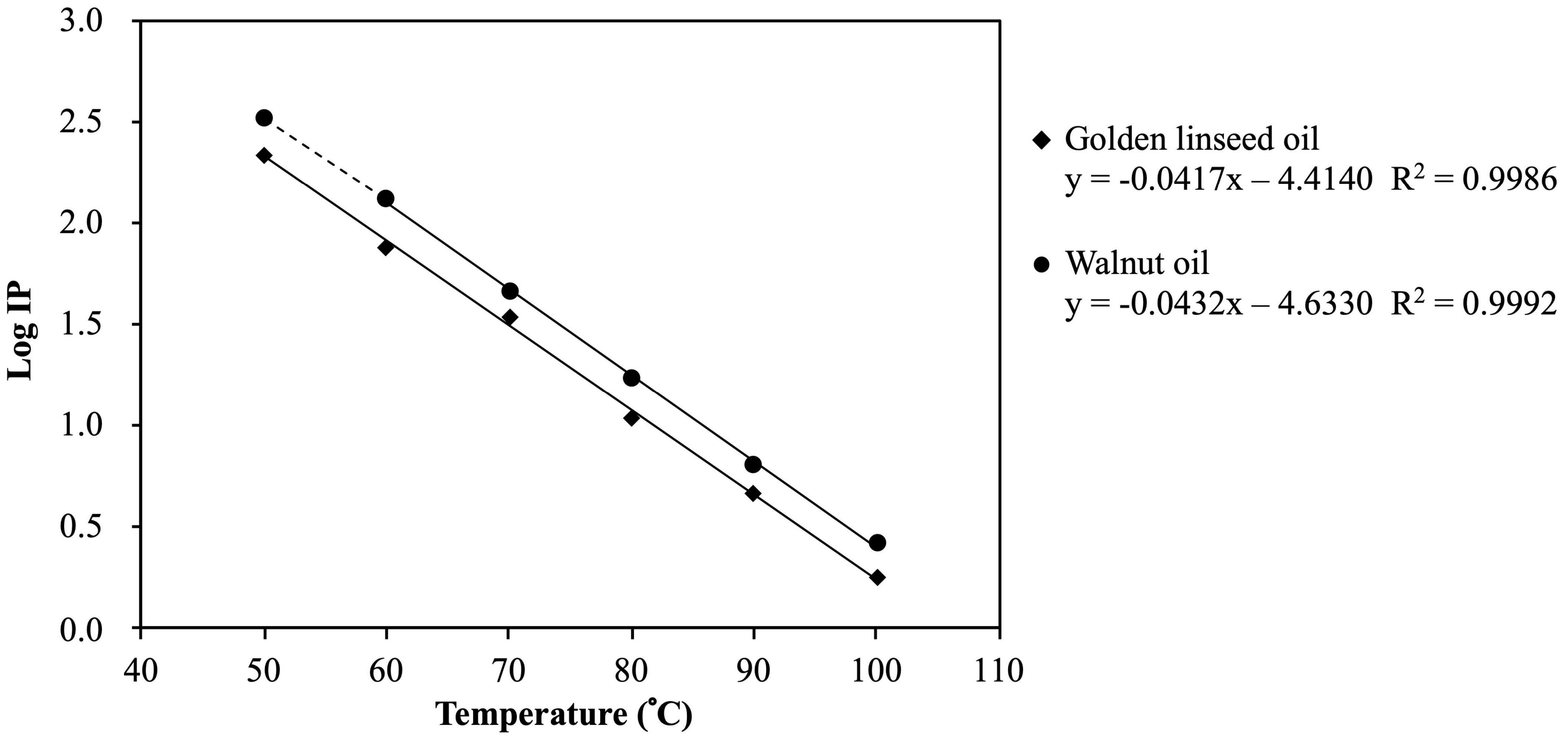

Supplementary Figure 2. Arrhenius plots in the temperature range of 50~100 ˚C. The IP value of the walnut oil sample at 50 ˚C was expressed as dotted line because it exceeded 240 h, the upper limit of OXITEST device
