# Supplementary material for: Application of OXITEST for Prediction of Shelf-Lives of Selected Cold-Pressed Oils
Source: Front Nutr. 2021 Oct 21;8:763524. doi: 10.3389/fnut.2021.763524 (PMC8566681; doi:10.3389/fnut.2021.763524)
Supplement: Supplementary file 2 [file Data_Sheet_2.PDF]

Supplementary Table 1. Recoveries and yields of ten expeller-pressed oils

Oil type	Oil recovery by solvent extraction (%)	Oil recovery by screw pressing (%)	Yield (%)[1]
Almond oil	43.5 ± 1.5[a]	40.9 ± 1.7[a]	94.0 ± 2.1
Black sesame oil	40.8 ± 1.0[a]	39.6 ± 2.3[a]	97.1 ± 5.0
Camellia oil	40.4 ± 2.2[a]	22.8 ± 2.8[b]	56.4 ± 4.7
Golden linseed oil	34.5 ± 1.2[a]	32.3 ± 1.1[a]	93.6 ± 0.9
Peanut oil	40.5 ± 0.9[a]	34.8 ± 1.2[b]	85.9 ± 5.8
Pecan oil	64.1 ± 0.7[a]	62.5 ± 3.3[a]	97.5 ± 5.0
Pine nut oil	50.6 ± 1.3[a]	36.9 ± 1.5[b]	72.9 ± 2.6
Pumpkin seed oil	35.5 ± 0.6[a]	37.1 ± 2.4[a]	104.5 ± 6.3
Sunflower seed oil	37.0 ± 1.3[a]	37.6 ± 2.3[a]	101.6 ± 3.3
Walnut oil	60.1 ± 2.8[a]	48.3 ± 11.4[a]	80.4 ± 19.2

Results are expressed as the mean ± standard deviation ($n = 3$).

[1] The yield (%) was calculated as a ratio of the oil content obtained by solvent extraction to that obtained by a screw press.

Different lowercase letters in the same row indicate a statistical difference ($p < 0.05$).

Supplementary Table 2. Contents of carotenoids and chlorophylls in ten expeller-pressed oils

**Oil type**	**Carotenoids (mg/kg)**	**Chlorophylls (mg/kg)**
Almond oil	0.95	0.49
Black sesame oil	3.54	0.36
Camellia oil	9.96	5.85
Golden linseed oil	6.02	0.62
Peanut oil	2.50	1.04
Pecan oil	5.72	0.21
Pine nut oil	10.08	0.07
Pumpkin seed oil	8.26	21.81
Sunflower seed oil	0.51	0.42
Walnut oil	1.51	0.68

Results are expressed as the mean (n = 2).

ND, not detectable

Supplementary Table 3. Tocopherol contents of nine expeller-pressed oils

**Oil type**	**α-Tocopherol (mg/100 g)**	**γ-Tocopherol (mg/100 g)**
Almond oil	37.8	10.1
Black sesame oil	ND	126.4
Camellia oil	17.8	15.2
Golden linseed oil	ND	60.3
Peanut oil	27.7	52.8
Pecan oil	ND	60.1
Pine nut oil	25.6	36.3
Sunflower seed oil	106.9	16.5
Walnut oil	ND	36.0

Results are expressed as the mean (n = 2).

ND, not detectable
